# Taraxasterol acetate targets RNF31 to inhibit RNF31/p53 axis-driven cell proliferation in colorectal cancer

**DOI:** 10.1038/s41420-021-00449-5

**Published:** 2021-04-06

**Authors:** Chao-Tao Tang, Jing Yang, Zi-De Liu, Youxiang Chen, Chunyan Zeng

**Affiliations:** Department of Gastroenterology, the First Affiliated Hospital of Nanchang University, Nanchang, China

**Keywords:** Drug development, Colon cancer

## Abstract

Colorectal cancer (CRC) is the third most common cancer worldwide. Several studies have suggested that taraxasterol acetate (TA) can inhibit the growth of tumor cells. However, to date, it remains unclear how TA inhibits cell growth and how RNF31 functions as an oncogene. We examined the expression of RNF31 in CRC tissue samples via immunohistochemistry and elucidated the function of RNF31 in CRC cells by constructing a cell model with RNF31 depletion. A cycloheximide (CHX)-chase analysis and immunofluorescence assays were conducted to demonstrate that TA can promote RNF31 degradation by activating autophagy. We used the PharmMapper website to predict targets of TA and identified RNF31. CHX-chase experiments showed that TA could facilitate RNF31 degradation, which was inhibited by the administration of chloroquine. Immunofluorescence assays showed that RNF31 protein was colocalized with LC3I/II and p62, suggesting that TA promoted RNF31 degradation by activating autophagy. We also found that CRC patients with RNF31 overexpression had poorer survival than those with low RNF31 expression. The results of the CHX-chase experiment showed that depletion of RNF31 alleviated p53 degradation, which was inhibited by MG132. A series of co-immunoprecipitation (Co-IP) assays revealed that RNF31 interacts with p53 and promotes p53 ubiquitination and degradation. A Co-IP assay performed with a truncated RNF31 plasmid showed that the PUB domain interacts with p53. Moreover, the PUB domain is the key structure in the induction of p53 ubiquitination. Our findings reveal a key role of RNF31 in CRC cell growth and indicate a mechanism through which TA inhibits cell growth.

## Introduction

Colorectal cancer (CRC) is one of the most common tumors. According to the latest statistics in 2018, CRC incidence ranked fourth, and the associated mortality rate ranked third^1^. At present, in China, among all malignant tumors, the incidence and mortality of CRC is ranked third and fifth, respectively, with an obvious increasing trend^2,3^. Three pathways are involved in the pathogenesis of sporadic CRC: the classic adenoma-adenocarcinoma pathway, the de novo pathway, and the colitis-associated carcinoma (CAC) pathway^4^. A series of genomic mutations or abnormal activation of molecular signaling pathways occur in the above processes, such as mutation or loss of the p53 gene, mutation of the KRAS gene and the APC gene, abnormal activation of the NF-κB signaling pathway, and abnormal autophagy in the intestinal epithelium and immune cells^4–7^. Over the past several decades, major advances in understanding CRC pathogenesis have been achieved; however, the prognosis of CRC patients remains poor due to high malignancy. Consequently, a better understanding of the molecular mechanisms underlying CRC would promote the development of effective therapeutic strategies for CRC patients.

Taraxasterol acetate (TA) is considered an essential component of dandelion, which is a type of Chinese medicine. Multiple studies have found that TA has a good inhibitory effect on inflammatory reactions. In rheumatoid arthritis, studies have found that TA can inhibit TNF-α, IL6, and IL8 expression and alleviate inflammation induced by IL-1β^8^. For hepatitis induced by concanavalin, TA alleviated liver injury by regulating the TLR/NF-κB and Bax/Bcl-2 cellular signaling axes^9^. Recently, Professor Che et al.^10^ found that treatment with TA in an AOM/DSS mouse model decreased the inflammatory response and that TA inhibited the release of IL6 and TNF-α in vitro, which suggested that TA may function as a preventive medicine for CRC. However, the effect of TA on CRC occurrence and development needs to be investigated. RNF31, a type of E3 ubiquitin ligase, is a member of the RING finger protein family and is also termed HOIL-1-interacting protein. Since RNF31 was initially found in breast cancer cells, it has been shown to be overexpressed in many tumors and associated with tumorigenesis, such as in breast cancer, pituitary adenoma and prostate cancer^11–13^. In addition, RNF31 has been reported to be involved in innate and adaptive immune responses. For instance, RNF31 can regulate atypical ubiquitination of FOXP2, which is a marker of T cells and is involved in self-tolerance and immune homeostasis^14^. In addition, RNF31 expression can stabilize some membrane receptors of B cells and tumor cells, such as estrogen receptor α and CD40^15–17^. Regarding the inflammatory response, RNF31 can attenuate TNF-α-induced NF-κB activation, which mediates cell apoptosis and the immune response of T cells^18^. Although RNF31 is considered a crucial mediator in the inflammatory response, the role of RNF31 in tumorigenesis is unknown, especially in CRC.

In the present study, through analysis of network pharmacology and prediction with the PharmMapper database, we found that RNF31 was a potential target of TA. Using in vitro assays, we elucidated the autophagic mechanism by which TA regulates RNF31 expression. Additionally, we found that RNF31 promoted CRC cell proliferation by enhancing p53 degradation via the ubiquitin–proteasome pathway.

## Results

### TA promoted RNF31 degradation by activating autophagy

**Fig. 1:**
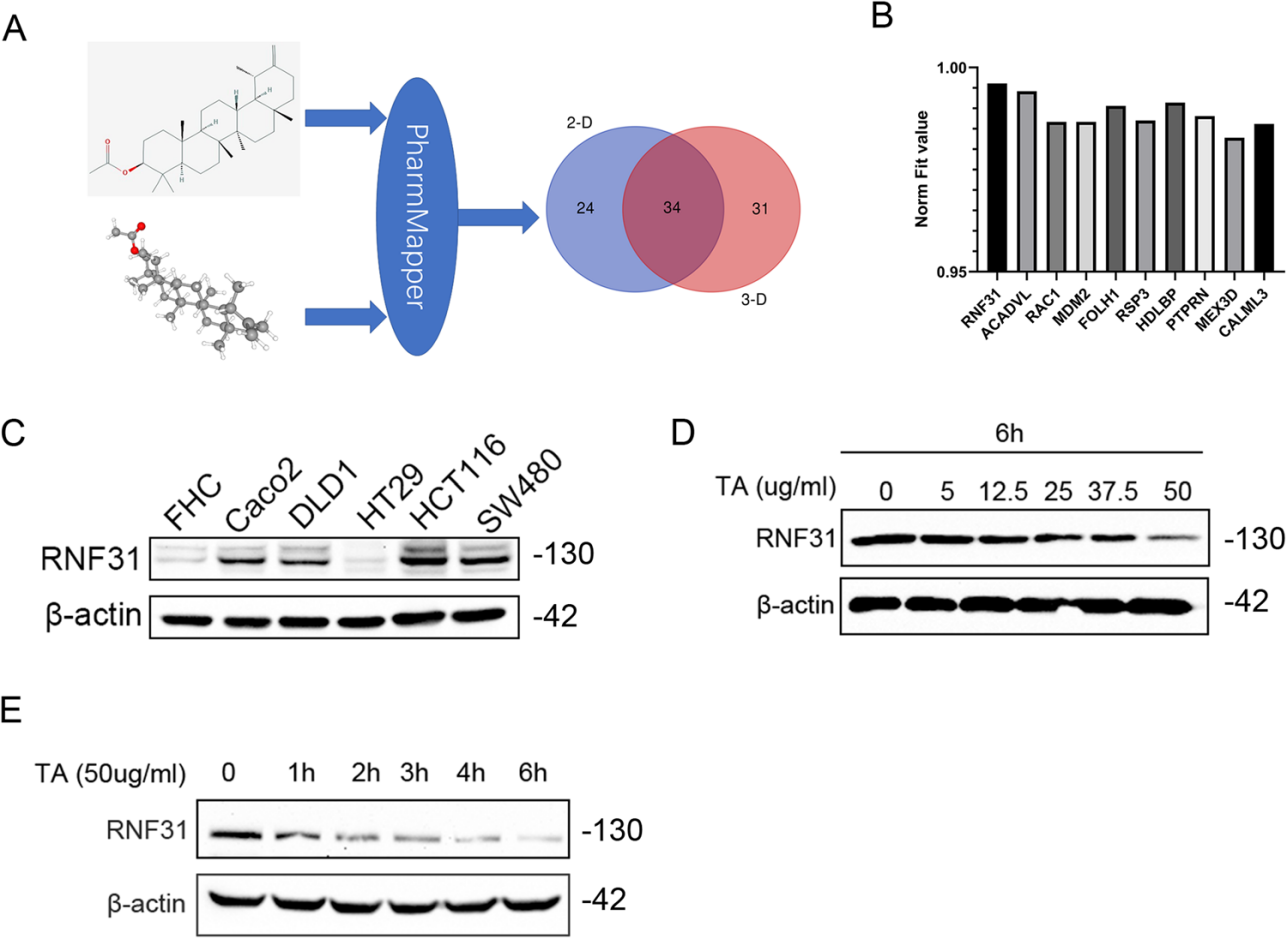
Identification of RNF31 as a target of TA. **A** In total, 34 potential targets were predicted by PharmMapper according to the 2D and 3D TA structures. **B** The top 10 proteins according to the Norm Fit value. **C** The basic RNF31 expression level in different CRC cell types. **D**, **E** Western blotting was performed to measure RNF31 expression after administration of TA at different concentrations or for different times.

**Fig. 2:**
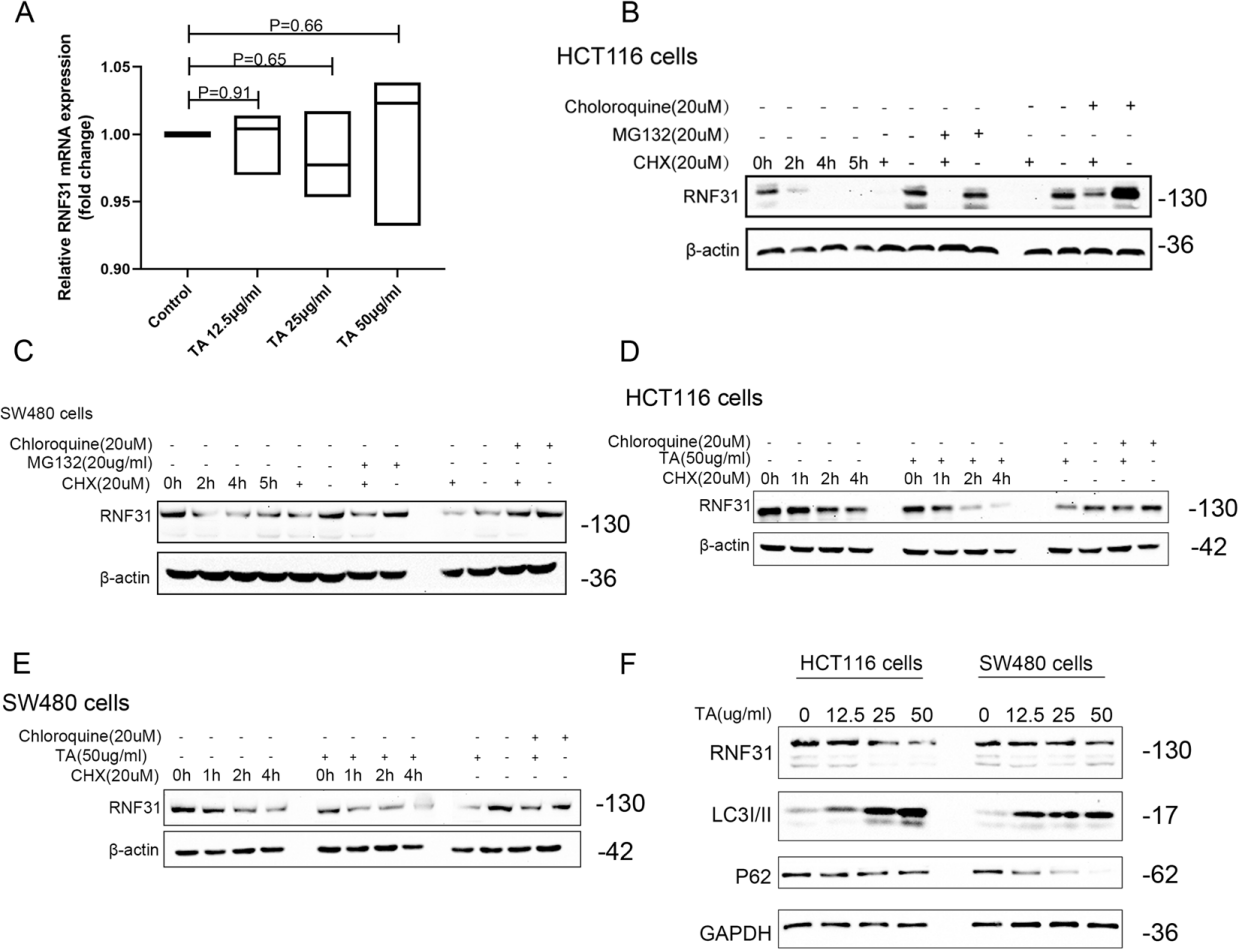
Autophagy activated by TA degraded RNF31 protein. **A** RT-PCR assays were performed to measure the RNF31 mRNA level after treatment with TA for 6 h at different concentrations. **B**, **C** Western blotting was performed to measure RNF31 protein degradation after treatment with CHX for different lengths of time and to compare the alteration in the RNF31 protein level after treatment with MG132 and treatment with chloroquine in HCT116 cells and SW480 cells, respectively. For CHX treatment, the first four lanes represent cells treated with 20 µM CHX for different numbers of hours, without MG132 or chloroquine application; the last eight lanes represent cells treated with 20 µM MG132 or 20 µM chloroquine accompanied by 20 µM CHX for 6 h. **D**, **E** Western blotting was performed to measure RNF31 protein degradation after treatment with CHX for different lengths of time or simultaneous treatment with CHX and TA for different lengths of time and to measure the alteration in the RNF31 protein level between single treatment with TA and combined treatment with chloroquine and TA in HCT116 cells and SW480 cells. The first four lanes represent cells treated with only 20 µM CHX for different numbers of hours; and the middle four lanes represent cells treated with 50 µg/ml TA and 20 µM CHX for different numbers of hours; the last four lanes represent cells treated with 50 µg/ml TA and 20 µM chloroquine for 6 h. **F** Western blotting was used to measure the alteration in the LC3I/II and p62 protein levels after administration of TA at different concentrations in HCT116 cells and SW480 cells.

**Fig. 3:**
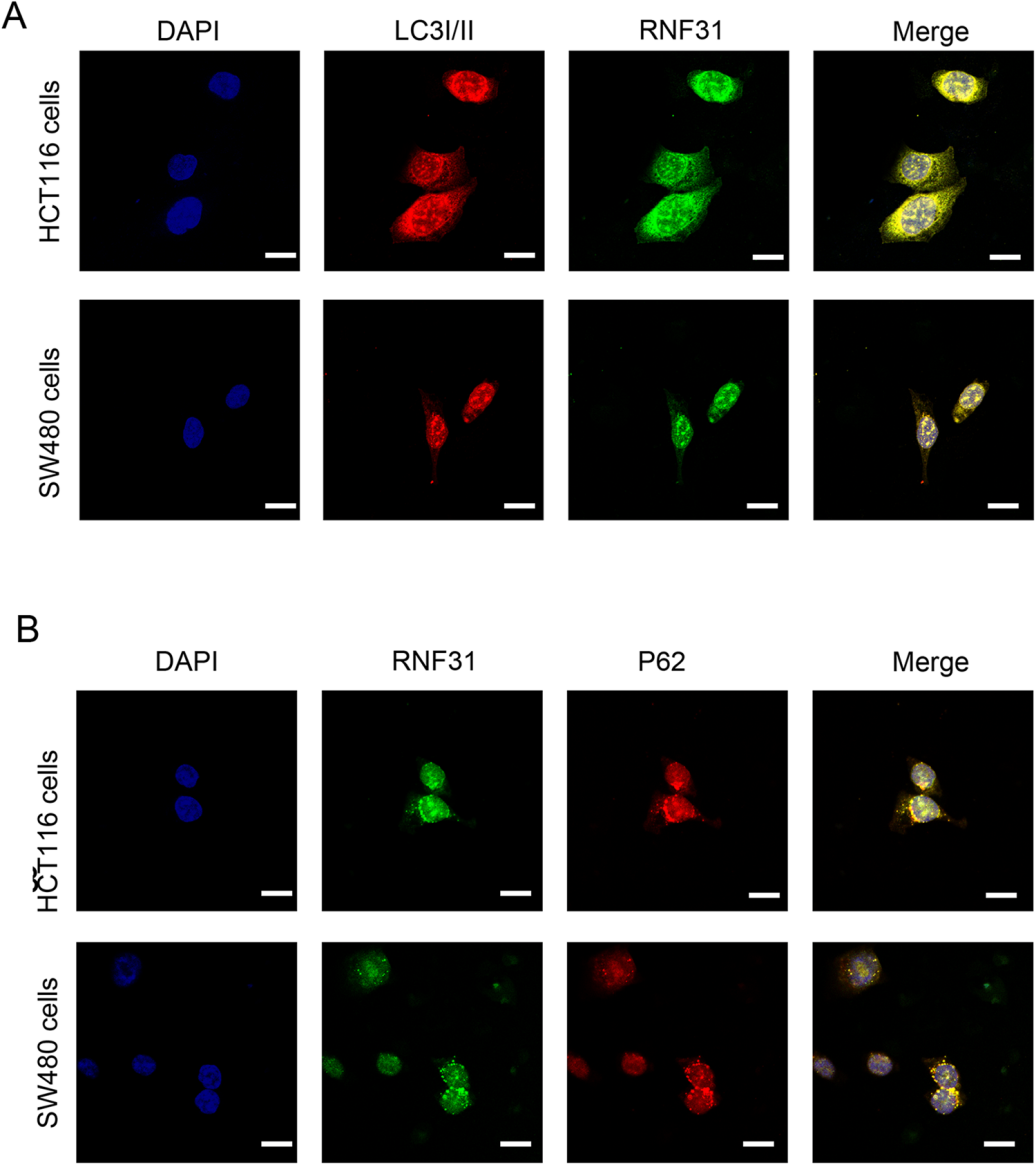
RNF31 colocalized with LC3I/II and p62. **A** Immunofluorescence assays were performed to identify colocalization between RNF31 and LC3I/II in HCT116 cells and SW480 cells. **B** Immunofluorescence assays were performed to identify colocalization between RNF31 and p62 in HCT116 cells and SW480 cells.

To investigate the function of TA, we used the PharmMapper website to identify potential targets. As shown in Fig. 1A, we predicted the targets according to the 2D and 3D structures of TA and found 34 proteins that were predicted by the two-structure types; then, we ranked the proteins according to the Norm Fit value and found that RNF31 had the highest value (Fig. 1B), suggesting that RNF31 may be a target protein of TA. Next, we measured the RNF31 expression level in different CRC cell lines and found that HCT116 and SW480 cells had higher RNF31 protein levels than the other cells (Fig. 1C). Hence, we performed a series of subsequent experiments with HCT116 cells and SW480 cells. To explore the effect of TA on RNF31 protein expression, we treated HCT116 cells with different concentrations of TA or the same concentration of TA for different lengths of time. The results suggested that the effect of TA on RNF31 protein expression was dose dependent and time dependent (Fig. 1D, E). To further explore the mechanism of RNF31 degradation induced by TA, we performed RT-PCR to measure the RNF31 mRNA level after treatment with TA at different concentrations. As shown in Fig. 2A, no significant difference was observed between the control group and the treatment group (*P* > 0.05). Subsequently, we treated CRC cells with cycloheximide (CHX) to inhibit protein synthesis. Assays of both cell lines showed that RNF31 protein was degraded over time, and this process could be inhibited by chloroquine rather than MG132, suggesting that RNF31 degradation may occur through the autophagic pathway (Fig. 2B, C). Moreover, we found that treatment with TA accelerated RNF31 degradation compared to treatment with CHX alone, and this effect was inhibited by chloroquine (Fig. 2D, E). Many studies have demonstrated that chloroquine is an autophagy inhibitor^19^. Hence, we proposed that TA activates the autophagic process. To verify this hypothesis, using Western blot assays, we measured the expression level of the LC3I/II and p62 proteins, which are markers of autophagy, after treatment with TA. In Fig. 2F, the results showed that the LC3I/II protein level was increased with TA treatment, while p62 protein expression was decreased, which suggests that TA activates autophagy. Additionally, we performed an immunofluorescence assay to identify RNF31 localization. We found that RNF31 was colocalized with LC3I/II and p62, which further demonstrated that RNF31 is correlated with autophagy (Fig. 3A, B).

### RNF31 overexpression predicted poor prognosis in CRC patients

**Fig. 4:**
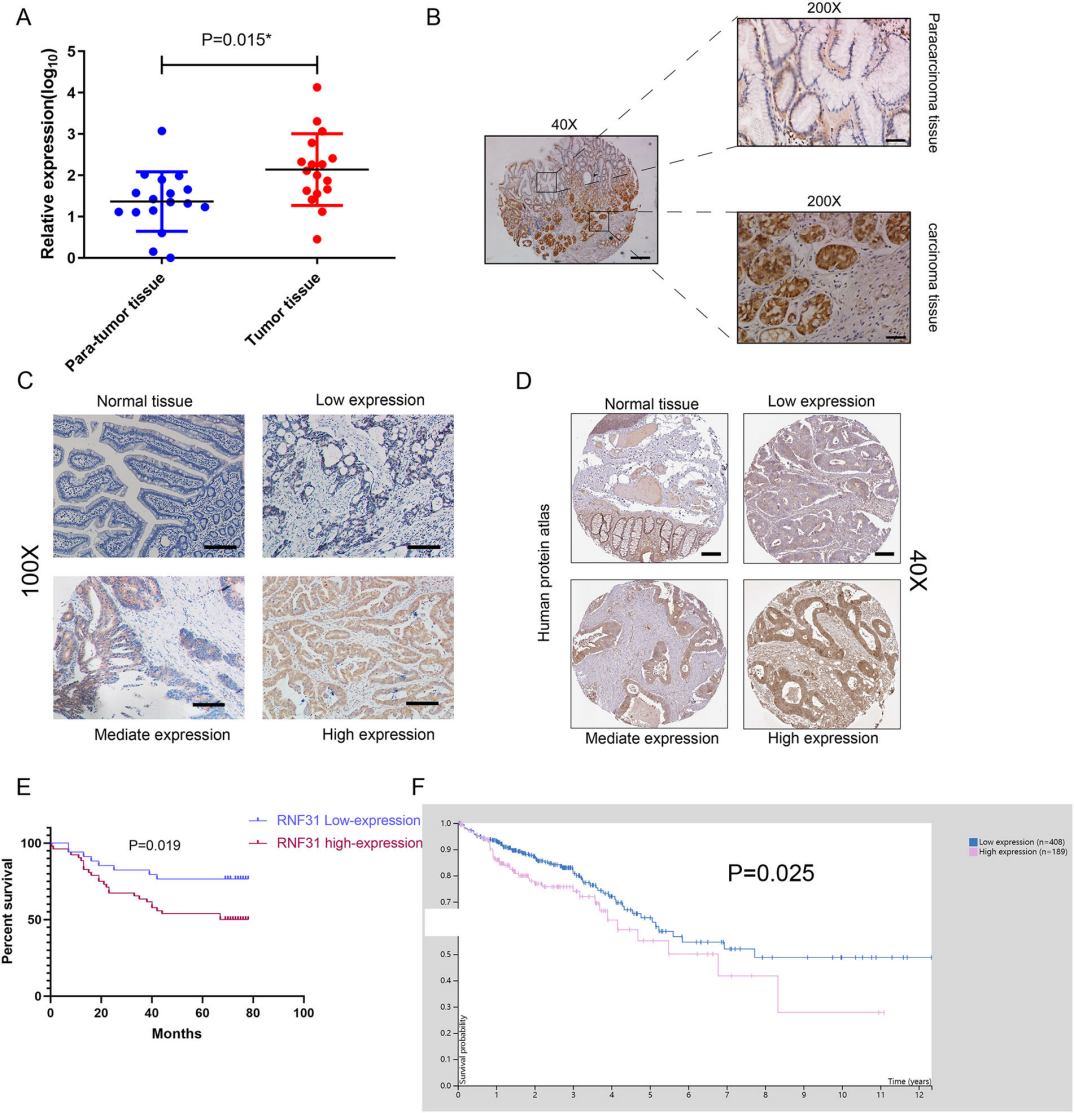
RNF31 overexpression predicted poor prognosis in patients with CRC. **A** The RNF31 mRNA level was measured via RT-PCR. **B** IHC assays were used to measure the RNF31 protein level in CRC tissue. **C** Representative images of different degrees of positive RNF31 expression in our patient tissues. **D** A representative image of different degrees of positive RNF31 expression in the Human Protein Atlas. **E** A K–M survival curve for our patients was constructed according to RNF31 expression. **F** A K–M survival curve was constructed according to RNF31 expression with information from patents from TCGA database.

Several studies have examined whether TA can inhibit cell proliferation and promote cell apoptosis^14,20,21^, and our studies indicated that RNF31 is a target of TA. Therefore, to investigate the effect of RNF31 on cell proliferation, we first measured the RNF31 expression level. As shown in Fig. 4A, we collected 17 pairs of CRC and para-carcinoma mucosal tissues via endoscopy. Then, we performed RT-PCR and found that RNF31 expression in the tumor tissue was higher than that in the para-carcinoma tissue. In addition, we collected surgical samples to perform IHC assays and similarly found that RNF31 protein was overexpressed in tumor tissues (Fig. 4B, C). Consistent with our results, the data provided by the Human Protein Atlas website showed that RNF31 was overexpressed in tumor tissues (Fig. 4D). To analyze the effect of RNF31 overexpression on prognosis, we performed a tissue microarray analysis of 86 pairs of CRC tissues with survival information and performed an IHC assay. According to the results of the IHC assay, we divided all patients into two groups, namely, an RNF31 overexpression group and RNF31 low-expression group (Supplementary Table 2). A chi-square test showed that RNF31 expression was associated with TNM stage and tumor size (*P* < 0.05). Univariate and multivariate Cox analyses revealed that RNF31 was an independent risk factor for prognosis, as well as lymph node metastasis, TNM stage, and distant metastasis (Table 1). A Kaplan–Meier (K–M) survival curve showed that RNF31 overexpression predicted poorer patient survival than low RNF31 expression (Fig. 4E), which was the same result found in The Cancer Genome Atlas (TCGA) database (Fig. 4F).

**Table 1:** Univariate and multivariate cox regression of prognostic factors for survival of patients with CRC.

Characteristic	Univariate analysis		Multivariate analysis	
	HR value	*P* value	HR value	*P* value
*Age*				
≤60	Reference	–		
>60	0.988(0.69–1.416)	0.949		
*Gender*				
Male	Reference	–		
Female	0.779(0.393–1.545)	0.475		
*TNM staging*				
I/II	Reference	–	Reference	–
III/IV	4.669(2.297–9.947)	*0.000*	3.547(2.077–8.765)	*0.000*
*Tumor type*				
Protuberant	Reference	–		
Infiltrative	3.505(1.077–11.41)	0.037		
Ulcerative	1.905(0.653–5.554)	0.238		
*Adenocarcinoma Pathological grade*				
I	Reference	–		
II	1.531(0.577–4.061)	0.392		
III	3.095(1.01–9.484)	0.048		
*Lymph node metastasis*				
No	Reference	–	Reference	*0.000*
Yes	4.237(2.111–8.507)	*0.000*	4.017(2.324–8.417)	
*Tumor size*				
≥5 cm	Reference	–	Reference	
<5 cm	1.823(0.902–3.685)	*0.045*	1.769(0.806–3.575)	0.078
*T stage*				
T1/T2	Reference	–		
T3/T4	2.357(0.568–9.095	0.236		
*Distant metastasis*				
No	Reference	–	Reference	
Yes	4.757(1.604–14.12)	*0.005*	4.307(1.459–13.075)	*0.008*
*RNF31 expression*				
Low	Reference	–	Reference	
High	2.458(1.112–5.443)	*0.026*	2.014(1.104–5.923)	*0.037*
*P53 expression*				
Low	Reference	–	Reference	
High	0.287(0.125–0.66)	*0.003*	0.351(0.145–0.776)	0.005

Italic values indicate statistical significance when *P* < 0.05.

### RNF31 promoted CRC cell proliferation by targeting the p53 signaling pathway

**Fig. 5:**
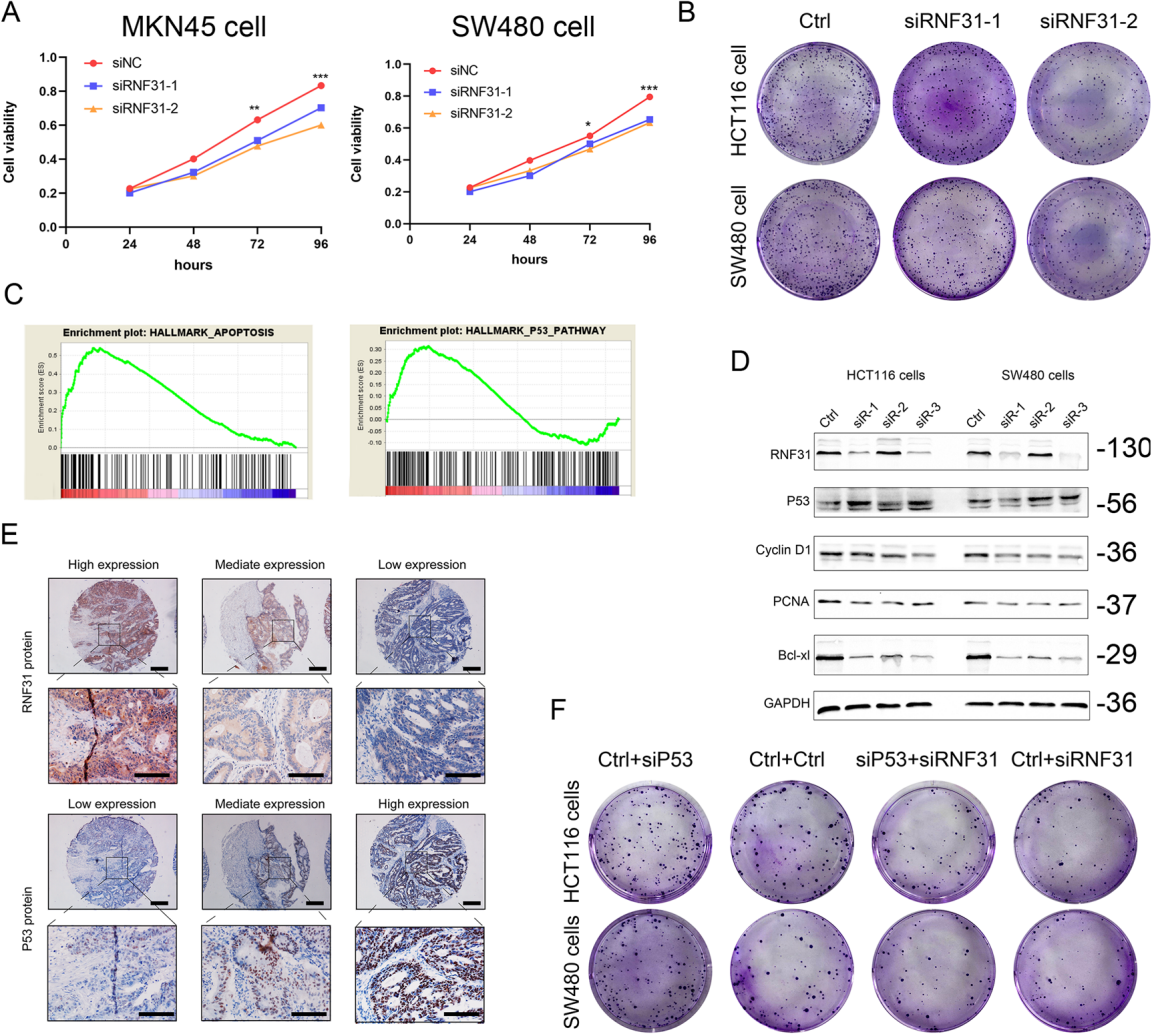
RNF31 promoted CRC cell growth via the p53 signaling pathway. **A** MTT assays were performed to assess alterations in cell viability after depletion of RNF31. **B** Cell colony formation assays were performed to assess alterations in cell viability after RNF31 depletion. **C** GSEA was performed to assess the effect of RNF31 on cell viability and to investigate downstream factors of RNF31. **D** Western blotting was performed to measure proteins related to cell growth, such as PCNA, Cyclin D1, and Bcl-xl, in HCT116 and SW480 cells. **E** The association of RNF31 with p53 in CRC tissues was assessed using IHC assays. **F** The alteration in cell viability was compared via cell colony formation assays after single depletion of RNF31 and simultaneous depletion of RNF31 and p53.

**Fig. 6:**
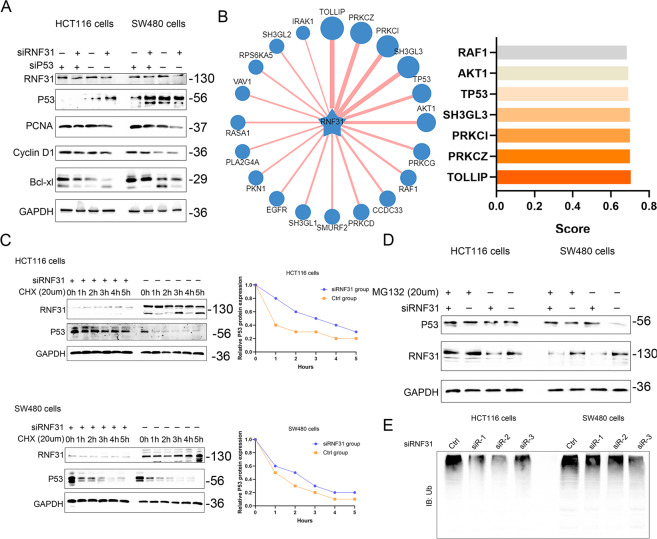
RNF31 promoted p53 degradation via the ubiquitin–proteasome pathway. **A** Western blot showing p53 expression and proteins related to cell growth, such as PCNA, Cyclin D1, and Bcl-xl, in HCT116 and SW480 cells after double depletion of p53 and RNF31 or single depletion of RNF31. **B** The Ubibrowser website was used to identify targets of RNF31. **C** A cycloheximide (CHX)-chase experiment was performed to compare the rate of p53 degradation between the RNF31 depletion group and the nonsense depletion group. The left picture shows p53 degradation measured by Western blotting, and the right picture shows the grayscale plot constructed using ImageJ software and GraphPad software. **D** Western blotting was used to assess the level of p53 when cells were treated with 20 µM MG132 and siRNF31. **E** Western blotting was conducted to measure the level of ubiquitin when RNF31 expression was depleted.

We constructed a cell model with RNF31 depletion and performed MTT and colony formation assays. As shown in Fig. 5A, B, we found that cell proliferation and colony formation were decreased when RNF31 expression was knocked down. Additionally, we performed GSEA using the TCGA database and found that RNF31 expression was correlated with cell apoptosis and that RNF31 was involved in the p53 signaling pathway (Fig. 5C). Consistent with the functional assays, Western blotting results revealed that cell proliferation-related proteins, such as PCNA and Cyclin D1, as well as the apoptosis-associated protein Bcl-xl, were downregulated after RNF31 depletion (Fig. 5D). Additionally, we found that p53 protein was upregulated when RNF31 protein was knocked down, suggesting that p53 expression is negatively associated with RNF31 expression (Fig. 5D). IHC results also showed that RNF31 protein expression was negatively correlated with p53 protein expression (Fig. 5E and Supplementary Table 2). In a rescue assay, we found that cell colony formation was obviously increased when p53 protein was depleted, and then, cells were simultaneously treated with p53 siRNA and RNF31 siRNA (Fig. 5F). Moreover, we found that some related proteins, such as PCNA, Cyclin D1, and Bcl-xl, exhibited corresponding changes consistent with the alterations in cell viability (Fig. 6A).

### RNF31 induced p53 ubiquitination and promoted its degradation via the proteasome pathway

**Fig. 7:**
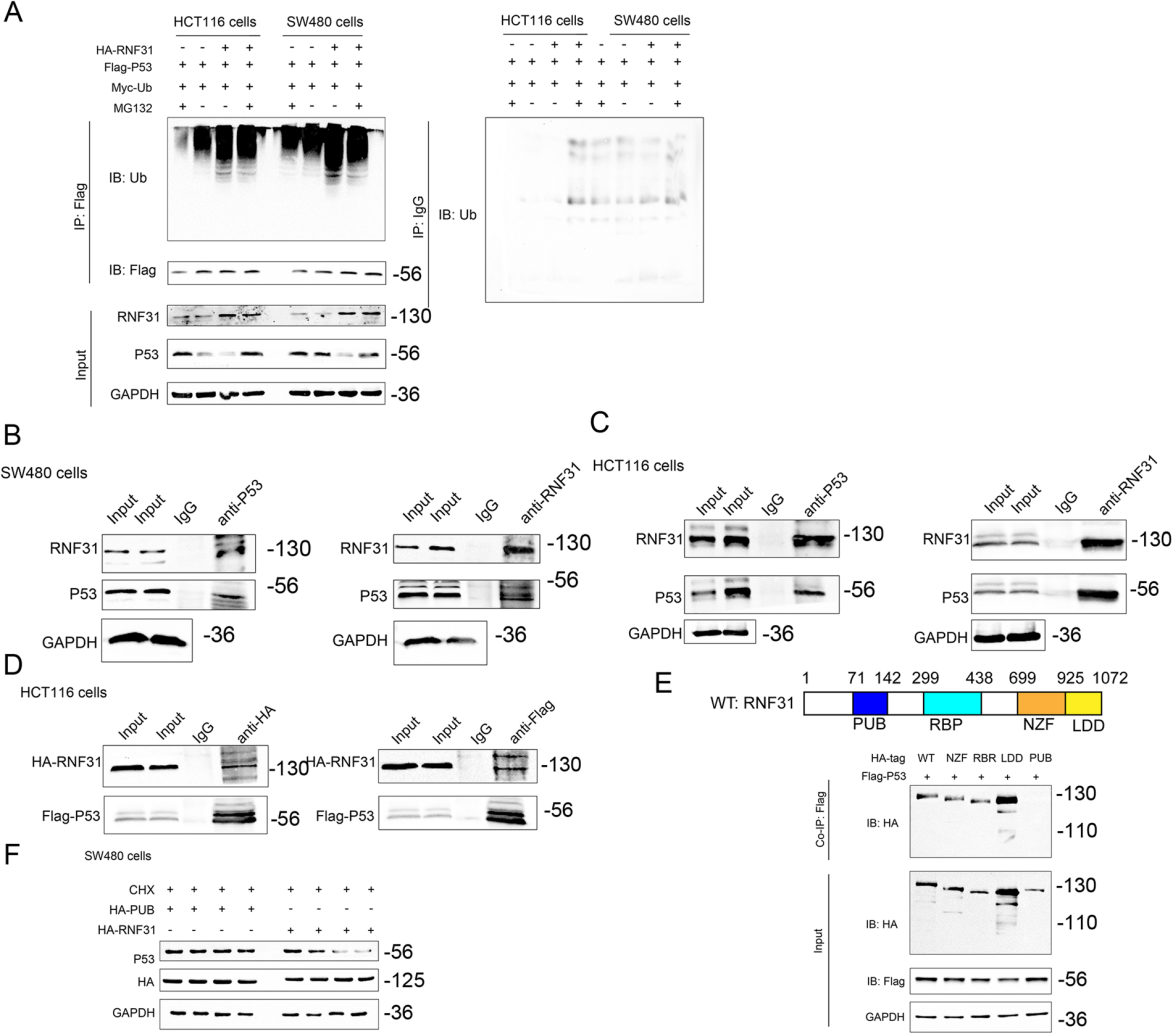
RNF31 interacted with p53 via the PUB domain. **A** HCT116 and SW480 cells were cotransfected with Myc-Ub, Flag-p53, and HA-RNF31 plasmids. After 48 h, the cells were treated with 20 µm MG132 for 6 h. The p53 ubiquitination level was assessed by immunoprecipitation, and p53 expression after treatment with MG132 was detected with Western blotting. **B**, **C** The interaction between endogenous RNF31 and p53 was detected by coimmunoprecipitation assays with anti-RNf31 and anti-p53 antibodies in HCT116 and SW480 cells. Immunoglobulin G (IgG) antibody was used as the control, and cell lysates were used to examine RNF31 and p53 expression. **D** The interaction between exogenous RNF31 and p53 was detected by coimmunoprecipitation assays with anti-RNF31 and anti-p53 antibodies in HCT116 cells. **E** SW480 cells were cotransfected with control vector, RNF31 WT plasmid, or truncated RNF31 mutants and Flag-p53 WT plasmid. A coimmunoprecipitation assay was performed to detect the interaction between RNF31 and p53 protein. **F** A cycloheximide (CHX)-chase experiment was performed to measure p53 degradation after transfection with HA-PUB plasmid and HA-RNF31 WT plasmid.

To further investigate the mechanism by which RNF31 regulates p53, we investigated the RNF31 structure and assessed whether RNF31 could affect the status of p53 ubiquitination. According to the prediction of the Ubibrowser website, p53 has high potential as an RNF31 substrate (Fig. 6B). In a CHX pulse-chase assay (Fig. 6C), we found that p53 protein was degraded over time without special treatment. Depletion of RNF31 expression enhanced p53 expression and slowed the degradation process, which was demonstrated in both HCT116 cells and SW480 cells. Additionally, treatment with MG132, a proteasome inhibitor, prevented p53 protein degradation and had an effect similar to that of RNF31 expression depletion (Fig. 6D). Consistent with the p53 protein degradation, knockdown of RNF31 decreased ubiquitin protein expression, suggesting that RNF31 regulates the status of ubiquitination (Fig. 6E). However, it remains unknown whether RNF31 expression is associated with ubiquitination of p53 protein. Hence, we measured the level of ubiquitin protein that was immunoprecipitated by HA-tagged antibody and compared the alteration in ubiquitin protein after treatment with RNF31 overexpression plasmid or with MG132 (Fig. 7A). The results suggested that overexpression of RNF31 induced p53 ubiquitination, which was inhibited by MG132. Additionally, the p53 protein level was negatively associated with the ubiquitination status. To further investigate the interaction between RNF31 and p53 protein, we performed a Co-IP assay using exogenous plasmid and truncated plasmid. As shown in Fig. 7B, C, we found that endogenous RNF31 protein interacted with endogenous p53 protein in both HCT116 cells and SW480 cells. Additionally, we found that RNF31 interacted with p53 protein (Fig. 7D). Considering that RNF31 protein has several domains, we designed several plasmids according to domain and performed a Co-IP assay, finding that mutation in the PUB domain resulted in loss of the interaction between RNF31 and p53 (Fig. 7E). Moreover, we found that transfection with the plasmid containing the PUB mutant did not produce results different from transfection with the wild-type RNF31 plasmid (Fig. 7F), suggesting that the PUB domain may be important for the function of RNF31 protein. Therefore, our results demonstrate that RNF31 promotes ubiquitination of p53 protein and that the key region may be the PUB domain.

### TA induced p53 expression upregulation and promoted interaction between RNF31 and p53 protein

**Fig. 8:**
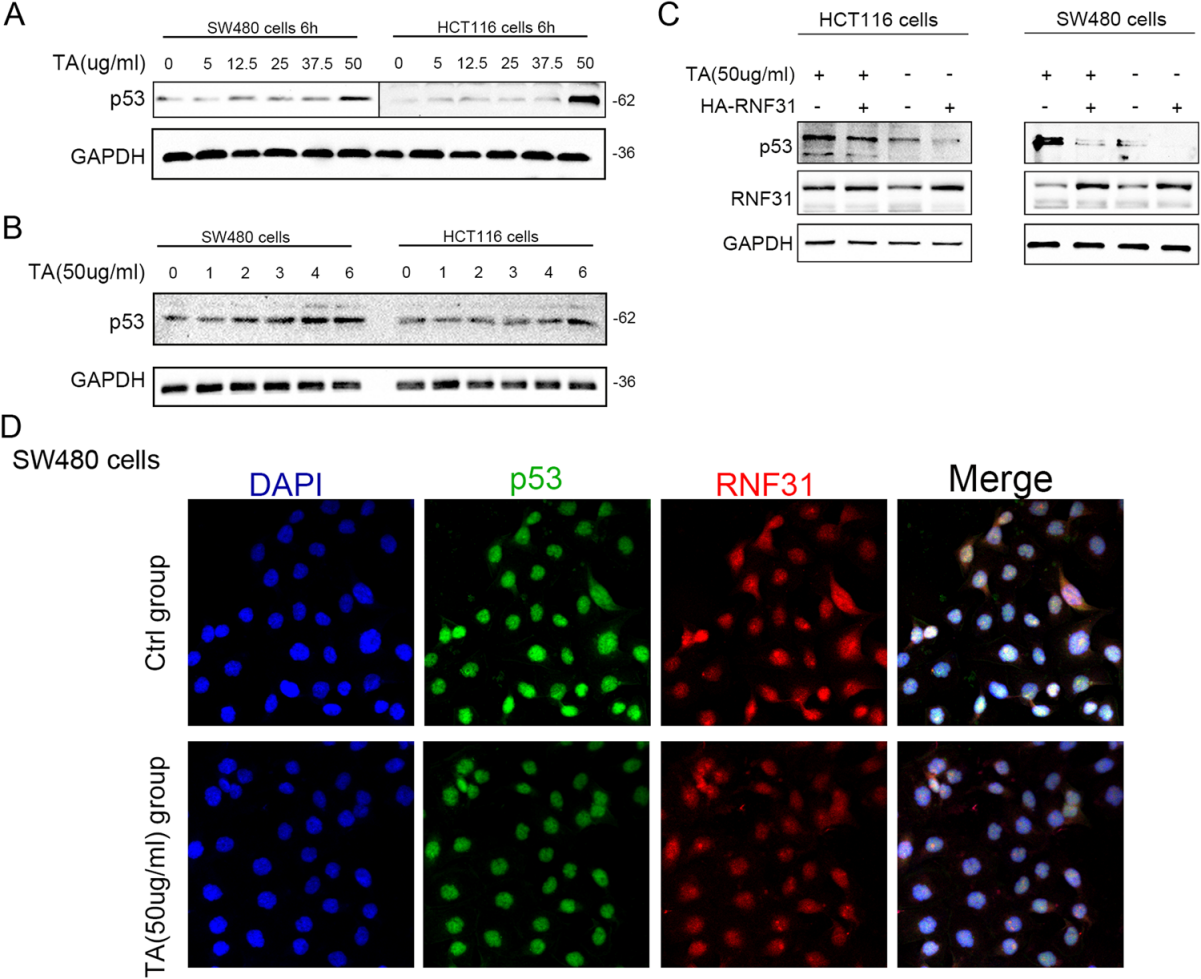
TA induced p53 protein overexpression and promoted the interaction of p53 with RNF31. **A** SW480 cells and HCT116 cells were treated with different concentrations for 6 h, and Western blotting was performed to measure the p53 protein level. **B** SW480 cells and HCT116 cells were treated with 50 µg/ml TA for different lengths of time, and then, Western blotting was performed to measure p53 protein expression. **C** SW480 and HCT116 cells were transfected with HA-RNF31 plasmid and treated with 50 µg/ml TA, and the alteration in p53 protein expression was measured. **D** Immunofluorescence assays were performed with SW480 cells, and the intensity of colocalization between p53 and RNF31 was compared after treatment of cells with 50 µg/ml TA.

Furthermore, we performed in vitro Western blotting and cell immunofluorescence assays to investigate the interaction between TA and p53. First, we treated HCT116 cells and SW480 cells with TA at different concentrations for 6 h or with 50 µg/ml TA for different numbers of hours. As shown in Fig. 8, we found that p53 protein expression was increased in a manner dependent on the TA concentration (Fig. 8A) or treatment time (Fig. 8B). Additionally, we upregulated RNF31 expression and treated the cells with TA at the same time and found that the increase in p53 protein induced by treatment with TA was downregulated due to RNF31 overexpression in HCT116 and SW480 cells (Fig. 8C). To evaluate changes in the interaction between p53 and RNF31, we performed immunofluorescence assays and found that the combined intensity between p53 and RNF31 protein was weakened after treatment with TA compared to the control group, suggesting that p53 degradation via the ubiquitin–proteasome system was reduced (Fig. 8D).

## Discussion

CRC is the third most common cancer type worldwide and has a 5-year survival rate of ~60%. Although most CRC cases are not derived from inflammatory bowel disease (IBD), the disease obviously increases the risk of CRC, especially for patients with a more than 5-year history of IBD^22,23^. Several studies have shown that TA has an anti-inflammatory effect in the development of colitis^24^, and the mechanism involves decreased release of inflammatory factors, such as TNF-α, IL-1β, and IL-6^25^. Moreover, accumulating evidence has shown that TA has potent antitumor activity against many tumor types, such as gastric cancer and hepatic cancer^26,27^. However, the effect of TA in CRC is unknown. In our study, we predicted the targets of TA via the PharmMapper tool and found that RNF31 protein was a potent target. Further in vitro studies demonstrated that RNF31 is degraded by autophagy induced by TA and is overexpressed in CRC to promote cell proliferation, with p53 as a downstream factor.

PharmMapper is a useful tool to investigate the mechanism of Chinese medicine^27,28^. Similarly, some studies have also predicted TA targets, such as EGFR and MMP2^24^, which differed from our results. Differences in methods and the diseases studied may explain the discrepancies between our study and previous studies. Earlier, Professor Che found that administration of TA could alleviate the inflammation induced by DSS in mice and that TA affected cell viability^10^. Regarding the inhibitory effect of TA on tumor cell proliferation, one study found that administration of TA in gastric cancer decreased EGFR and AKT1 expression, while another study found that TA suppressed the growth of liver cancer cells by upregulating nucleotide-binding protein 1, which is a tumor suppressor^26,27^. Interestingly, we predicted a new target protein of TA, RNF31, and found that treatment with TA decreased RNF31 expression. For the mechanism, we found that administration of chloroquine inhibited the effect of TA on RNF31, suggesting that the autophagic pathway degraded RNF31 protein. A recent study found that RNF31 regulates autophagic initiation and that RNF31 protein is colocalized with LC3I/II protein, which was consistent with our results and those of another study^29,30^.

With regard to the downstream factors of RNF31, we performed GSEA, which has been used in many studies, and found that p53 protein is a potent signaling molecule^31^. Previous studies have also found that the colonic mucosa in a group treated with TA exhibited higher p53 expression^10^. In the colonic mucosa of patients with ulcerative colitis, p53 protein expression was closely associated with the status of the disease^23^. In acute inflammation, a high level of infiltrating leukocytes is related to low p53 expression; however, dysplasia arises with increased p53 expression^23^. Therefore, the p53 signaling pathway plays an important role in the development of colitis-associated cancer. In our study, we found that degradation by the ubiquitin–proteasome system was the mechanism by which RNF31 regulates p53 protein, which was consistent with previous studies. As a member of the linear ubiquitin chain assembly complex (LUBAC), RNF31 has been considered to be responsible for the ubiquitination of proteins. For instance, RNF31 can stabilize FOXP3 by promoting conjugation of the atypical ubiquitin chain to FOXP3, resulting in the immune activity of T cells^14^. Similarly, RNF31 can stabilize estrogen receptor α by catalyzing atypical ubiquitination^32^. However, our study demonstrated that RNF31 promotes p53 degradation by catalyzing ubiquitination, which was different from the results of other studies. Actually, as a type of RBR E3 ubiquitin ligase, RNF31 can promote ubiquitination through two opposite roles, namely, atypical ubiquitination and typical ubiquitination; atypical ubiquitination stabilizes proteins, while typical ubiquitination leads to protein degradation^14,20^. Although one study on breast cancer also found that RNF31 facilitates p53 ubiquitination, our study has many highlights^12^. For example, we are the first to identify the negative association of RNF31 with p53 in clinical mucosal tissue. Regarding the interaction between RNF31 and p53, we further found that the PUB domain of RNF31 interacts with p53 and that mutation of PUB in RNF31 protein results in an inability to degrade p53.

## Conclusions

In summary, our work reports the indispensable and positive role of RNF31 in maintaining CRC cell growth as an oncogene, uncovers the underlying mechanism of TA in mediating RNF31 expression and finds that RNF31 catalyzes p53 protein ubiquitination via the PUB domain.

## Methods

### Patients and tissue samples

In our study, two types of colorectal mucosal tissue were used: tissues obtained by surgery and tissues obtained by endoscopy. We used 86 pairs of surgical tissues to generate a tissue microarray after formalin fixation and paraffin embedding (HColA180Su19, purchased from Outdo Biotech Company, Shanghai, China) and conducted an immunohistochemistry (IHC) analysis. Sixteen pairs of endoscopic mucosal tissues obtained from the First Affiliated Hospital of Nanchang University were used to perform real-time quantitative PCR to measure RNF31 mRNA levels. All patients provided informed consent forms. Our study was approved by the Ethics Committee of the First Affiliated Hospital of Nanchang University. Detailed information on the patients who underwent surgery is shown in Supplementary Table 2.

### Bioinformatics analysis

To investigate potential target proteins of TA, we used the PharmMapper website (http://www.lilab-ecust.cn/pharmmapper/). First, we found the 2D and 3D structures of TA reported by Sigma-Aldrich. Then, we uploaded the file into the PharmMapper website and selected the correct parameters according to the introduction. Finally, the results were exported. To identify the downstream RNF31 signaling pathway proteins, we performed gene set enrichment analysis (GSEA) with R software. Moreover, we used Ubibrowser (http://ubibrowser.ncpsb.org/) to identify ubiquitinated proteins induced by RNF31.

### Cell culture and transfection

HEK293T, HCT116, and SW480 cells were purchased from American Type Culture Collection. The cell lines were cultured as previously described^33^. Briefly, CRC cells were cultured in RPMI-1640 medium (Gibco Company, USA) mixed with 10% fetal bovine serum (FBS), 100 U/ml penicillin, and 50 mg/ml streptomycin. The cell incubator conditions were 37 °C with a humidified atmosphere containing 5% CO_2_. Cell transfection was performed using Lipofectamine™ 2000 (Invitrogen, Carlsbad, CA, USA), and cotransfection was performed with FuGENE HD (Promega) according to the manufacturer’s instructions. The siRNA and plasmid sequences are shown in Supplementary Table 1.

### Cell proliferation (MTT) and colony formation assays

To perform MTT assays and colony formation assays, we used 96-well plates and 6-well plates, respectively. For the MTT assays, CRC cells were incubated in plates at a density of 10^3^ cells per well. The next day, the cells were transfected with RNF31 siRNA or RNF31 overexpression plasmid. Then, cell viability was measured at 490 nm after incubation with MTT reagent for 4 h at 37 °C. For colony formation assays, 1000 cells were seeded in cell plates and then treated with siRNA or plasmid. Ten days later, cell colonies in the plates were fixed with formaldehyde and stained with 1% crystal violet. Finally, the number of colonies was determined using Photoshop software.

### Western blot analysis

All protein was extracted in radioimmunoprecipitation assay (RIPA) buffer (Solarbio Life Science, Beijing, China) mixed with protease inhibitor on ice-cold plates. Then, the protein concentration was determined with a bicinchoninic acid (BCA) protein assay kit purchased from Solarbio Life Science. Next, we loaded equal amounts of protein into a 10% SDS–PAGE gel for separation and transferred the protein onto nitrocellulose membranes (Millipore). The membranes were blocked with 5% BSA (Sigma-Aldrich) and incubated with primary antibodies at 4 °C overnight. On the second day, the membranes were washed with 1× TBS solution and incubated with secondary antibody conjugated with horseradish peroxidase (HRP) at normal temperature for 1 h. Finally, the results were obtained using a ChemiDoc^TM^ Imaging System (Bio-Rad, Hercules, CA, USA). Throughout the entire study, primary antibodies targeting the following proteins were used: RNF31 (ab46332, 1:1000, Abcam), p53 (ab1101, 1:1000 Abcam), PCNA (ab29, 1:1000, Abcam), Cyclin D1 (ab40754, 1:5000, Abcam), Bcl-xl (2764, 1:1000, CST), GAPDH (1:1000, TransGen Biotech, Beijing), Flag-tag (D6W5B, CST), HA-tag (C29F4, CST), and β-actin (1:1000, TransGen Biotech, Beijing). The secondary antibodies were purchased from TransGen Biotech (Beijing).

### Coimmunoprecipitation (Co-IP)

A Co-IP experiment was performed to determine whether RNF31 interacts with p53 protein. CRC cells were seeded in 10-cm plates and then scraped with RIPA lysis buffer. A volume of 1 μL of primary antibody, 50 μL of Protein A-Agarose (sc-2003; Santa Cruz Biotechnology, Dallas, TX, USA), and 500 μL of phosphate-buffered saline (PBS) was mixed and incubated together for 2 h. Next, the cellular proteins were incubated with primary antibody overnight at 4 °C on a rocking platform. Primary antibodies targeting RNF31, p53, and IgG (BL003A, Bio-Sharp, Shanghai, China) were used. The next day, the cells were centrifuged at 3000 rpm at 4 °C and washed twice with PBS containing protein inhibitors, followed by Western blotting with the primary antibodies used for the Co-IP experiments.

### Immunohistochemistry

CRC tissue was warmed at 70 °C for 2 h, dewaxed with xylene and anhydrous ethanol for 40 min, and incubated in citrate to complete antigen retrieval. Finally, tissue microarrays were covered with primary antibody overnight at 4 °C. On the second day, the microarrays were incubated with the corresponding secondary antibody for 30 min at room temperature. After the samples were washed with PBS, the tissue was stained with DAB reagent (TransGen Biotech, Beijing), and the nuclei were stained with hematoxylin. The IHC results were assessed using a method described previously^33^. In short, protein expression was assessed according to the intensity of staining and the percentage of positively stained cells. The results were divided into three groups: high expression (scores 7–9), moderate expression (scores 4–7), and low expression (scores 0–3), which were blindly and independently assessed by pathologists.

### Cell immunofluorescence assay

CRC cells were incubated in chamber slides for 24 h, fixed in 4% paraformaldehyde for 20 min, and permeabilized with 0.1% Triton X-100 for 1 h. Next, cells were incubated with primary antibodies targeting RNF31 (MAB8039, R&D Systems) and LC3I/II (3868, CST) or p62 (23214, CST) overnight at 4 °C. Finally, the cells were incubated with goat anti-rabbit or anti-mouse IgG fluorescent secondary antibody (Thermo Fisher Scientific), and the nuclei were stained with DAPI. Immunofluorescence intensity was examined via confocal fluorescence microscopy at wavelengths of 488 and 594 nm.

### Real-time quantitative PCR

Total RNA from tissues was lysed with TRIzol reagent (TransGen Biotech, Beijing) and extracted with chloroform and isopropyl alcohol. Then, the RNA concentration was measured with a NanoDrop 2000 spectrophotometer (Thermo Scientific, Wilmington, DE, USA). cDNA synthesis was completed according to the instructions of reagents purchased from TransGen Biotech. Similarly, quantitative PCR was performed with the reagents (TransGen Biotech). The RNF31 primer sequences were as follows: forward 5′–3′ ACTGGCGTGGTGTCAAGTTTA and reverse 5′–3′ CGGGGAAGCTCAACCCATC. The GAPDH primer sequences were the same as previously reported^33^.

### Statistical analysis

As described in previous studies, most statistical analyses were performed with SPSS and GraphPad Prism 8 software. A chi-squared test was used to investigate the relationship between RNF31 expression and clinicopathological factors. Univariate and multivariate Cox regression analyses were used to identify factors related to prognosis. Survival curves associated with RNF31 and p53 expression were plotted using the K–M method and compared using a log-rank test. A two-tailed Student’s *t*-test was used to assess differences between the control group and treatment group. All assays were repeated more than twice. Differences were considered to be statistically significant when *P* < 0.05.

## Supplementary information

Supplementary table 1

Supplementary table 2
