# Supplementary material for: Taraxasterol acetate targets RNF31 to inhibit RNF31/p53 axis-driven cell proliferation in colorectal cancer
Source: Cell Death Discov. 2021 Apr 6;7:66. doi: 10.1038/s41420-021-00449-5 (PMC8024285; doi:10.1038/s41420-021-00449-5)
Supplement: Supplementary file 1 — Supplementary table 1 [file 41420_2021_449_MOESM1_ESM.docx]

**Supplementary table 1: The Sequence of RNF31 siRNA and** **The Sequence of RNF31 truncated plasmids**

| RNF31 siRNA sequence | 5’——3’ | 5’——3’ |
| --- | --- | --- |
| 1 | CGUGGUGUCAAGUUUAAUATT | UAUUAAACUUGACACCACGTT |
| 2 | CCUAUGCGUUGUUCCAUAATT | UUAUGGAACAACGCAUAGGTT |
| 3 | CCUGUGCCAUGCUAAAUGATT | UCAUUUAGCAUGGCACAGGTT |

**RNF31 mutation of RBR**

Atgccgggggaggaagaggagcgggccttcctggtggcccgcgaggagctggcgagcgccctgaggagggattccgggcaggcgttttccctggagcagctccggccgctactagccagctctctgccgctagccgcccgctacctgcagctggacgccgcacgccttgtccgctgcaacgctcatggggagccccgaaactacctcaacaccctgtccacggctctgaacatcctggagaaatacggccgcaaccttctcagccctcagcggcctcggtactggcgtggtgtcaagtttaataaccctgtctttcgcagcacggtggatgctgtgcaggggggccgagatgtgctgcgattatatggctacacagaggagcaaccagatgggttgagcttccccgaagggcaggaggagccagatgagcaccaggttgctacagtcacactggaagtactgctgcttcggacagagctcagcctgctattgcagaatactcatccaagacagcaggcactggagcagctgttggaagacaaggttgaagatgatatgctgcagctttcagaatttgaccccctattgagagagattgctcctggccccctcaccacaccctctgtcccaggctccactcctggtccctgcttcctctgtggttctgccccaggcacactgcactgcccatcctgtaaacaggccctgtgtccagcctgtgaccacctgttccatggacacccatcccgtgctcatcacctccgccagaccctgcctggggtcctgcagggtacccacctgagccccagtttacctgcctcagcccaaccacggccccagtcgacctccctgctggccctgggagacagctctct

**RNF31 dPUB sequence**

atgccgggggaggaagaggagcgggccttcctggtggcccgcgaggagctggcgagcgccctgaggagggattccgggcaggcgttttccctggagcagctccggccgctactagccagctctctgccgctagccgcccgctacctgcagctggacgccgcacgccttgtccgctgcaacgctcatggggagccccgaaactacctcaacgagcaccaggttgctacagtcacactggaagtactgctgcttcggacagagctcagcctgctattgcagaatactcatccaagacagcaggcactggagcagctgttggaagacaaggttgaagatgatatgctgcagctttcagaatttgaccccctattgagagagattgctcctggccccctcaccacaccctctgtcccaggctccactcctggtccctgcttcctctgtggttctgccccaggcacactgcactgcccatcctgtaaacaggccctgtgtccagcctgtgaccacctgttccatggacacccatcccgtgctcatcacctccgccagaccctgcctggggtcctgcagggtacccacctgagccccagtttacctgcctcagcccaaccacggccccagtcgacctccctgctggccctgggagacagctctctttcttcccctaatcctgcaagtgctcatttgccctggcactgtgctgcctgtgccatgctaaatgagccttgggcagtgctctgtgtggcctgtgatcggccccgaggctgtaaggggttggggttgggaactgagggtccccaaggaactggaggcctagaacctgatcttgcacggggtcggtgggcctgccagagctgtacctttgagaatgaggcagctgctgtgctatgttccatatgtgagcgacctcggctggcccagcctcccagcttggtggtggattcccgagatgctggcatttgcctgcaaccccttcagcagggggatgctttgctggcctctgcccagagtcaagtctggtactgtattcactgtaccttctgcaactcgagccctggctgggtgtgtgttatgtgcaaccggactagtagccccattccagcacaacatgccccccggccctatgccagctctttggaaaagggaccccccaagcctgggcccccacgacgccttagtgcccccctgcccagttcctgtggagatcctgagaagcagcgccaagacaagatgcgggaagaaggcctccagctagtgagcatgatccgggaaggggaagccgcaggtgcctgtccagaggagatcttctcggctctgcagtactcgggcactgaggtgcctctgcagtggttgcgctcagaactgccctacgtcctggagatggtggctgagctggctggacagcaggaccctgggctgggtgccttttcctgtcaggaggcccggagagcctggctggatcgtcatggcaaccttgatgaagctgtggaggagtgtgtgaggaccaggcgaaggaaggtgcaggagctccagtctctaggctttgggcctgaggaggggtctctccaggcattgttccagcacggaggtgatgtgtcacgggccctgactgagctacagcgccaacgcctagagcccttccgccagcgcctctgggacagtggccctgagcccaccccttcctgggatgggccagacaagcagagcctggtcaggcggcttttggcagtctacgcactccccagctggggccgggcagagctggcactgtcactgctgcaggagacacccaggaactatgagttgggggatgtggtagaagctgtgaggcacagccaggaccgggccttcctgcgccgcttgcttgcccaggagtgtgccgtgtgtggctgggccctgccccacaaccggatgcaggccctgacttcctgtgagtgcaccatctgtcctgactgcttccgccagcacttcaccatcgccttgaaggagaagcacatcacagacatggtgtgccctgcctgtggccgccccgacctcaccgatga

**RNF31 dLDD sequence**

atgccgggggaggaagaggagcgggccttcctggtggcccgcgaggagctggcgagcgccctgaggagggattccgggcaggcgttttccctggagcagctccggccgctactagccagctctctgccgctagccgcccgctacctgcagctggacgccgcacgccttgtccgctgcaacgctcatggggagccccgaaactacctcaacaccctgtccacggctctgaacatcctggagaaatacggccgcaaccttctcagccctcagcggcctcggtactggcgtggtgtcaagtttaataaccctgtctttcgcagcacggtggatgctgtgcaggggggccgagatgtgctgcgattatatggctacacagaggagcaaccagatgggttgagcttccccgaagggcaggaggagccagatgagcaccaggttgctacagtcacactggaagtactgctgcttcggacagagctcagcctgctattgcagaatactcatccaagacagcaggcactggagcagctgttggaagacaaggttgaagatgatatgctgcagctttcagaatttgaccccctattgagagagattgctcctggccccctcaccacaccctctgtcccaggctccactcctggtccctgcttcctctgtggttctgccccaggcacactgcactgcccatcctgtaaacaggccctgtgtccagcctgtgaccacctgttccatggacacccatcccgtgctcatcacctccgccagaccctgcctggggtcctgcagggtacccacctgagccccagtttacctgcctcagcccaaccacggccccagtcgacctccctgctggccctgggagacagctctctttcttcccctaatcctgcaagtgctcatttgccctggcactgtgctgcctgtgccatgctaaatgagccttgggcagtgctctgtgtggcctgtgatcggccccgaggctgtaaggggttggggttgggaactgagggtccccaaggaactggaggcctagaacctgatcttgcacggggtcggtgggcctgccagagctgtacctttgagaatgaggcagctgctgtgctatgttccatatgtgagcgacctcggctggcccagcctcccagcttggtggtggattcccgagatgctggcatttgcctgcaaccccttcagcagggggatgctttgctggcctctgcccagagtcaagtctggtactgtattcactgtaccttctgcaactcgagccctggctgggtgtgtgttatgtgcaaccggactagtagccccattccagcacaacatgccccccggccctatgccagctctttggaaaagggaccccccaagcctgggcccccacgacgccttagtgcccccctgcccagttcctgtggagatcctgagaagcagcgccaagacaagatgcgggaagaaggcctccagctagtgagcatgatccgggaaggggaagccgcaggtgcctgtccagaggagatcttctcggctctgcagtactcgggcactgaggtgcctctgcagtggttgcgctcagaactgccctacgtcctggagatggtggctgagctggctggacagcaggaccctgggctgggtgccttttcctgtcaggaggcccggagagcctggctggatcgtcatggcaaccttgatgaagctgtggaggagtgtgtgaggaccaggcgaaggaaggtgcaggagctccagtctctaggctttgggcctgaggaggggtctctccaggcattgttccagcacggaggtgatgtgtcacgggccctgactgagctacagcgccaacgcctagagcccttccgccagcgcctctgggacagtggccctgagcccaccccttcctgggatgggccagacaagcagagcctggtcaggcggcttttggcagtctacgcactccccagctggggccgggcagagctggcactgtcactgctgcaggagacacccaggaactatgagttgggggatgtggtagaagctgtgag

**RNF31 dNZF sequence**

atgccgggggaggaagaggagcgggccttcctggtggcccgcgaggagctggcgagcgccctgaggagggattccgggcaggcgttttccctggagcagctccggccgctactagccagctctctgccgctagccgcccgctacctgcagctggacgccgcacgccttgtccgctgcaacgctcatggggagccccgaaactacctcaacaccctgtccacggctctgaacatcctggagaaatacggccgcaaccttctcagccctcagcggcctcggtactggcgtggtgtcaagtttaataaccctgtctttcgcagcacggtggatgctgtgcaggggggccgagatgtgctgcgattatatggctacacagaggagcaaccagatgggttgagcttccccgaagggcaggaggagccagatgagcaccaggttgctacagtcacactggaagtactgctgcttcggacagagctcagcctgctattgcagaatactcatccaagacagcaggcactggagcagctgttggaagacaaggttgaagatgatatgctgcagctttcagaatttgaccccctattgagagagattgctcctggccccctcaccacaccctctgtcccaggctccactcctggtccctgcttcctctgtggttctgccccaggcacactgcactgcccatcctgtaaacaggccctgtgtccagcctgtgaccacctgttccatggacacccatcccgtgctcatcacctccgccagaccctgcctggggtcctgcagggtacccacctgagccccagtttacctgcctcagcccaaccacggccccagtcgacctccctgctggccctgggagacagctctctttcttcccctaatcctgcaattccagcacaacatgccccccggccctatgccagctctttggaaaagggaccccccaagcctgggcccccacgacgccttagtgcccccctgcccagttcctgtggagatcctgagaagcagcgccaagacaagatgcgggaagaaggcctccagctagtgagcatgatccgggaaggggaagccgcaggtgcctgtccagaggagatcttctcggctctgcagtactcgggcactgaggtgcctctgcagtggttgcgctcagaactgccctacgtcctggagatggtggctgagctggctggacagcaggaccctgggctgggtgccttttcctgtcaggaggcccggagagcctggctggatcgtcatggcaaccttgatgaagctgtggaggagtgtgtgaggaccaggcgaaggaaggtgcaggagctccagtctctaggctttgggcctgaggaggggtctctccaggcattgttccagcacggaggtgatgtgtcacgggccctgactgagctacagcgccaacgcctagagcccttccgccagcgcctctgggacagtggccctgagcccaccccttcctgggatgggccagacaagcagagcctggtcaggcggcttttggcagtctacgcactccccagctggggccgggcagagctggcactgtcactgctgcaggagacacccaggaactatgagttgggggatgtggtagaagctgtgaggcacagccaggaccgggccttcctgcgccgcttgcttgcccaggagtgtgccgtgtgtggctgggccctgccccacaaccggatgcaggccctgacttcctgtgagtgcaccatctgtcctgactgcttccgccagcacttcaccatcgccttgaaggagaagcacatcacagacatggtgtgccctgcctgtggccgccccgacctcaccgatgacacacagttgctcagctacttctctacccttgacatccagcttcgcgagagcctagagccagatgcctatgcgttgttccataagaagctgaccgagggtgtgctgatgcgggaccccaagttcttgtggtgtgcccagtgctcctttggcttcatatatgagcgtgagcagctggaggcaacttgtccccagtgtcaccagac
