# Supplementary material for: Taraxasterol acetate targets RNF31 to inhibit RNF31/p53 axis-driven cell proliferation in colorectal cancer
Source: Cell Death Discov. 2021 Apr 6;7:66. doi: 10.1038/s41420-021-00449-5 (PMC8024285; doi:10.1038/s41420-021-00449-5)
Supplement: Supplementary file 2 — Supplementary table 2 [file 41420_2021_449_MOESM2_ESM.docx]

| **Supplementary Table 2**: Correlation of RNF31 expression with the clinicopathological characteristics of CRC | | | | |
| --- | --- | --- | --- | --- |
| RNF31 expression | | | | |
| Characteristic | Category | Low  (n=34,%) | High  (n=52%) | *P* value |
| Age | ≤60  >60 | 5  29 | 23  29 | *0.004* |
| Gender | Male  Femal | 20  14 | 26  26 | 0.422 |
| TNM staging | I/II  III/IV | 26  8 | 27  25 | *0.022* |
| Tumor location | Colon  Rectum | 27  7 | 47  5 | 0.151 |
| Tumor type | Protuberant  Infiltrative | 4  3 | 3  5 | 0.608 |
|  | Ulcerative | 27 | 44 |  |
| Adenocarcinoma Pathological grade | I | 9 | 9 | 0.463 |
|  | II | 21 | 33 |  |
|  | III | 4 | 10 |  |
| Lymph node metastasis | No  Yes | 25  9 | 29  23 | 0.095 |
| Tumor size | ≥5cm  <5cm | 13  21 | 32  20 | *0.034* |
| T stage | T1/T2  T3/T4 | 5  29 | 5  47 | 0.471 |
| Distant metastasis | No  Yes | 32  2 | 50  2 | 0.303 |
| P53 expression | Low | 15 | 35 | *0.031* |
|  | High | 19 | 17 |  |

Italic values indicate statistical significance when *P<0.05*
